# Attributes and definitions of locomotor capacity in older people: a World Health Organisation (WHO) locomotor capacity working group meeting report

**DOI:** 10.1007/s40520-022-02080-5

**Published:** 2022-02-08

**Authors:** Nicola Veronese, Germain Honvo, Jotheeswaran Amuthavalli Thiyagarajan, René Rizzoli, Cyrus Cooper, Olivier Bruyère, Christopher Mikton, Yuka Sumi, Theresa Diaz, Jean-Yves Reginster, Anshu Banerjee, the members of the WHO Collaborating Center Working Group on Locomotor Capacity, Al- Daghri Nasser, Andrieu Sandrine, Annweiler Cédric, Aubertin- Leheudre Mylène, Bautmans Ivan, Beaudart Charlotte, Becker Clemens, Bruyère Olivier, Buckinx Fanny, Campusano Claudia, Cesari Matteo, Chandran Manju, Cherubini Antonio, Clark Patricia, Cooper Cyrus, Cruz- Jentoft Alfonso, Dennison Elaine, Fouasson Chailloux Alban, Fuggle Nick, Gichu Muthoni, Gielen Evelien, Guicheux Jérôme, Harvey Nick, Haugen Ida, Honvo Germain, Lamy Olivier, Landi Francesco, Lane Nancy, Lazaretti Castro Marise, Lewiecki Mike, Matijevic Radmila, Mkinsi Ouafa, Mobasheri Ali, Njeze Ngozi, Pinto Daniel, Reginster Jean-Yves, Rizzoli René, Rolland Yves, Saleh Yousef, Singer Andrea, Thomas Thierry, Van der Velde Nathalie, Vellas Bruno, Veronese Nicola, Visser Marjolein, Zee A Han

**Affiliations:** 1 Geriatric Unit, Department of Medicine, University of Palermo, Palermo, Italy; 2 Ageing and Health Unit, Department of Maternal, Newborn, Child and Adolescent Health & Ageing, WHO HQ, Geneva, Switzerland; 3 World Health Organization Collaborating Centre for Public Health Aspects of Musculoskeletal Health and Aging, Liege, Belgium; 4 Division of Public Health, Epidemiology and Health Economics, University of Liège, Liège, Belgium; 5 Division of Bone Diseases, Faculty of Medicine, Geneva University Hospitals, University of Geneva, Geneva, Switzerland; 6 MRC Life Course Epidemiology Unit, Southampton General Hospital, University of Southampton, Southampton, UK; 7 Demographic Change and Healthy Aging Unit, Social Determinants of Health, World Health Organization, Geneva, Switzerland; 8 Epidemiology, Monitoring and Evaluation Unit, Maternal, Newborn, Child, Adolescent Health and Ageing, World Health Organization, Geneva, Switzerland

**Keywords:** Locomotor capacity, Older people, Attributes, Conceptual definition

The United Nations (UN) has proclaimed 2021–2030 the Decade of Healthy Ageing, with the World Health Organization (WHO) leading international action to improve the lives of older people, their families, and communities. The Decade brings together a variety of stakeholders for ten years of concerted action to (a) change how we think, feel and act towards age and ageing; (b) develop communities in ways that foster the abilities of older people; (c) deliver person-centred, integrated care and primary health services that are responsive to older people; and (d) provide older people access to long-term care when they need it [1, 2].

Healthy ageing [3] is defined by the World Health Organization (WHO) as “the process of developing and maintaining the functional ability that enables wellbeing in older age” [4, 5]. Functional ability comprises the health-related attributes that enable people to be and to do what they have reason to value. It is made up of the intrinsic capacity of the individual, relevant environmental characteristics, and the interactions between the individual and these characteristics. Intrinsic capacity is the composite of all the physical and mental capacities of an individual. To assess the impact of its actions at the national, regional, and global levels, the WHO proposed intrinsic capacity and functional ability as outcome indicators of healthy ageing. WHO’s integrated care for older people (ICOPE) framework suggests locomotor capacity as one of the domains of intrinsic physical capacity [6].

Locomotor capacity is broadly described as a person’s bodily capacity or physical capacity to move from one place to another [2, 7]. Four main characteristics proposed in a recent publication include a function of joints, bones, reflexes and muscle strength [2]. Other attributes (such as balance, endurance, muscle power) of the musculoskeletal system are also crucial in assessing locomotor capacity in older persons. While there is some clarity on the structure of intrinsic capacity and its subdomains, clear and consensual conceptual and operational definitions of locomotor capacity are still lacking.

To close these gaps, the WHO Collaborating Center for Public Health Aspects of Musculoskeletal Health and Aging (Liège, Belgium), in collaboration with the WHO Ageing and Health Unit, held a virtual Working Group meeting (see Supplementary Material for the participants), convening experts from around the world, to discuss the conceptual and operational definitions of locomotor capacity. The purpose of this Working Group was to provide WHO with clear conceptual and operational definitions of locomotor capacity, to enable the monitoring and evaluation of the impact of public health actions taken at the national, regional, and global levels to improve the lives of older people, their families, and communities, within the framework of the UN’s Decade of Healthy Ageing initiative.

Briefly, the most important points discussed by the experts during this initial Working Group were:

- Potential issues: locomotor capacity is often described as a person’s bodily capacity or physical capacity to move from one place to another. Although this implies the evaluation of mechanisms involved in the movement, there are three main issues with the current description discussed in the meeting: (a) movement of a person from one location to another would imply moving inside the home and from home to different locations in the community (e.g. park); this overlaps with the ability to be mobile, which is the domain of functional ability, (b) the description does not specify all the necessary attributes that should be considered for measurement, and (c) it did not clearly state whether it is a function of the body or a function of a person in the environment as the term bodily capacity was not clearly described.
- Mobility vs locomotor capacity: differently, mobility is a domain of functional ability that captures how a potential (locomotor capacity) is actualized in real life when interacting with the environment. Locomotor capacity is a domain of intrinsic capacity and mainly relates to the individual’s body function, whereas mobility reflects the interaction between an individual and his environment.
- Defining mobility*:* it is essential to define mobility better before defining the concept of locomotor capacity. Both concepts have some aspects in common. For instance, in some WHO documents, gait speed is a measure of locomotor capacity, but, for example, asking older persons whether they can walk 100 m is considered a measure of mobility. The WHO representatives emphasize that another Working group on mobility is currently working on the conceptual and operational definitions of the ability to be mobile.
- Attributes of locomotor capacity: experts agreed that locomotor capacity could be defined using at least the following attributes: endurance, balance, muscle strength, muscle function, muscle power and joint function of the body.
- Assessment of locomotor capacity: experts suggested that the term ‘capacity’ refers to the highest possible level of functioning in a given domain at a given moment in time. Consequently, an assessment tool would require a test that puts the particular locomotor aspect under stress. In this regard, some discussion emerged regarding whether locomotor capacity could be captured by performance-based tests or reported directly by older people.

In conclusion, based on the discussion above, the Working Group proposed a conceptual working definition that defines *locomotor capacity* as “a state (static or dynamic over time) of the musculoskeletal system that encompasses endurance, balance, muscle strength, muscle function, muscle power and a joint function of the body”. A systematic review of measures of locomotor capacity will be undertaken in the coming months to develop operational definitions for monitoring and evaluation of public health actions focusing on older persons. Further meetings and works are planned to elaborate the operational definition of locomotor capacity.

## Supplementary Information

Below is the link to the electronic supplementary material.

Supplementary file1 (DOCX 16 KB)
