# Supplementary material for: Attributes and definitions of locomotor capacity in older people: a World Health Organisation (WHO) locomotor capacity working group meeting report
Source: Aging Clin Exp Res. 2022 Feb 8;34(3):481–3. doi: 10.1007/s40520-022-02080-5 (PMC8894172; doi:10.1007/s40520-022-02080-5)
Supplement: Supplementary file 1 — Supplementary file1 (DOCX 16 KB) [file 40520_2022_2080_MOESM1_ESM.docx]

*Invited Experts and Co-authors (name, academic affiliation, country)*

1. Al-Daghri Nasser, College of Science and Chair for Biomarkers of Chronic Diseases, King Saud University, Riyadh, Saudi Arabia.
2. Andrieu Sandrine, Clinical epidemiology and public health department, Gerontopôle, Toulouse University Hospital, France
3. Annweiler Cédric, Department of Geriatric Medicine, University of Angers, France
4. Aubertin-Leheudre Mylène, Exercise sciences department, Université du Québec à Montréal (UQAM), Canada
5. Bautmans Ivan, Gerontology and Frailty in Ageing research departments, Vrije Universiteit Brussel (VUB), Belgium
6. Beaudart Charlotte, Department of Health Services Research, Maastricht University, Maastricht, The Netherlands; WHO Collaborating Center for Public Health Aspects of Musculoskeletal Health and Ageing, University of Liège, Belgium
7. Becker Clemens, Digital Geriatric Medicine, University of Heidelberg, Germany
8. Bruyère Olivier, WHO Collaborating Center for Public Health Aspects of Musculoskeletal Health and Ageing, University of Liège, Belgium
9. Buckinx Fanny, WHO Collaborating Center for Public Health Aspects of Musculoskeletal Health and Ageing, University of Liège, Belgium
10. Campusano Claudia, Universidad de los Andes, Chile
11. Cesari Matteo, Geriatric Fellowship Program, University of Milan, Italy
12. Chandran Manju, Osteoporosis and Bone Metabolism Unit, Singapore General Hospital, Singapore
13. Cherubini Antonio, Geriatria, Accettazione geriatrica e Centro di ricerca per l’invecchiamento, IRCCS INRCA, Ancona, Italy,
14. Clark Patricia, Clinical Epidemiology Research Unit, National University of México UNAM, Mexico
15. Cooper Cyrus, MRC Lifecourse Epidemiology Unit, University of Southampton, UK
16. Cruz-Jentoft Alfonso, Geriatric Department, Hospital Universitario Ramón y Cajal, Madrid, Spain
17. Dennison Elaine, MRC Lifecourse Epidemiology Unit, University of Southampton, UK
18. Fouasson Chailloux Alban, "Regenerative Medicine and Skeleton" research centre, University Hospital of Nantes, France
19. Fuggle Nick, MRC Lifecourse Epidemiology Center, University of Southampton, UK
20. Gichu Muthoni, Ministry of Health Kenya, Division of Geriatric Medicine, Kenya
21. Gielen Evelien, Unit of Gerontology and Geriatrics, Department of Public Health and Primary Care, KU Leuven, Belgium
22. Guicheux Jérôme, "Regenerative Medicine and Skeleton" research centre, University of Nantes, France
23. Harvey Nick, MRC Lifecourse Epidemiology Centre, University of Southampton, UK
24. Haugen Ida, Division of Rheumatology and Research, Diakonhjemmet Hospital, Norway
25. Honvo Germain, WHO Collaborating Center for Public Health Aspects of Musculoskeletal Health and Ageing, University of Liège, Belgium
26. Lamy Olivier, Bone Unit, Lausanne University Hospital, Switzerland
27. Landi Francesco, Geriatric Internal Medicine Unit, A. Gemelli University Hospital, Rome, Italy
28. Lane Nancy, Davis School of Medicine in Saccramento, University of California, USA
29. Lazaretti Castro Marise, Bone and Mineral Research Unit, Federal University of Sao Paulo (UNIFESP), Brazil
30. Lewiecki Mike, Bone Health TeleECHO, University of New Mexico Health Sciences Center in Albuquerque, New Mexico, USA
31. Matijevic Radmila, Rehabilitation Unit, Orthopaedic and Trauma, University of Novi Sad, Serbia
32. Messina Osvaldo Daniel, Rheumatology, C Argerich Hospital, University of Buenos Aires, Argentina
33. Mkinsi Ouafa, Department of Rheumatology, Ibn Rochd University Hospital, Casablanca, Morocco
34. Mobasheri Ali, Research Unit of Medical Imaging, Physics and Technology, University of Oulu, Finland
35. Njeze Ngozi, University of Nigeria Medical school, Nsukka, Nigeria
36. Pinto Daniel, Department of Physical Therapy, Marquette University, USA
37. Reginster Jean-Yves, WHO Collaborating Centre for Public Health Aspects of Musculoskeletal Health and Aging, University of Liège, Belgium
38. Rizzoli René, Faculty of Medicine, Geneva University Hospitals, Geneva, Switzerland
39. Rolland Yves, Gérontopôle of Toulouse, University Toulouse III Paul Sabatier, France
40. Saleh Yousef, College of Medicine, King Saud Bin Abdulaziz University for Health Sciences, Riyadh, Saudi Arabia
41. Singer Andrea, Division of Women’s Primary Care, MedStar Georgetown University Hospital, USA
42. Thomas Thierry, Rheumatology Department, University Hospital of Saint-Étienne (UHSE), France
43. Van der Velde Nathalie, Amsterdam UMC, The Netherlands
44. Vellas Bruno, Gérontopôle & Department of Geriatric Internal Medicine, Toulouse University Hospital, France
45. Veronese Nicola, Geriatric Unit, Department of Medicine, University of Palermo, Italy
46. Visser Marjolein, Vrije Universiteit Amsterdam, The Netherlands
47. Zee A Han, College of Medicine, The Catholic University of Korea, Seoul, South-Korea
